# The New Combustible

**Published:** 1874-03

**Authors:** 


### THE NEW COMBUSTIBLE.

We stated, says *Galignani's Messenger*, a short time ago, that a Belgian peasant had made the extraordinary discovery that earth, coal and soda mixed up together would burn as well and better than any other combustible, and the fact has since been proved beyond a doubt. The way in which he found this out is curious. He had been scraping the floor of his cellar with a shovel in order to bring all the bits of coal lying about into a heap, which, mixed as it was with earth and other impurities, he put into his stove. To his astonishment he found that this accidental compound burnt better instead of worse than he expected, and emitted much greater heat. Being an intelligent man he endeavored to discover the cause, and found that a great deal of soda, probably the remnant of the last wash, lay about on the floor of the cellar, and that some of it must have got into his heap. He then made a few experiments, and at length improved his compound sufficiently to render it practical. The publicity given in Belgium to this discovery caused trials to be made everywhere, and it has now been ascertained that three parts of the earth and one of coal dust, watered with a concentrated solution of soda, will burn well and emit great heat. Many Parisian papers talked of it, but only one—the *Moniteur*—went so far as to make the experiment at its printing office. A certain quantity of friable and slightly sandy earth was mixed with the quantum of coal dust prescribed; the two ingredients were well incorporated with each other, and then made into a paste with the solution above mentioned. The fireplace of one of the boilers had previously been lighted with coal, and the fire was kept up with shovelfuls of the mixture. The latter, in a few seconds, was transformed into a dry, brown crust, which soon after became red hot and then burned brightly, but without being very rapidly consumed. The fact of the combustion is, therefore, well ascertained; but before the system can be universally adopted there are some important points to be considered, such as the calorific power of the mixture compared with that of pure coal, its price, and, above all, a remedy for the great drawback attaching to it—its fouling the fire grate considerably.

